# Lower Extremity Abscess Formation in Premature Infants due to Routine Infant Vaccinations

**DOI:** 10.1155/2017/3290184

**Published:** 2017-06-18

**Authors:** Yuhang Sun, Surya N. Mundluru, Alice Chu

**Affiliations:** NYU Hospital for Joint Diseases, New York, NY 10003, USA

## Abstract

Since the introduction of vaccines, the impact of vaccinations has been immeasurable. Under the current immunization guidelines, infants receive the first of their routine infant vaccinations at 2 months of age. While the benefits of routine infant vaccinations in premature infants have been demonstrated, there is relatively little data on the dosing of these vaccines in premature infants. The medical records of two premature infants who developed intramuscular abscesses after receiving their routine infant vaccinations were reviewed. Both patients developed pain in the area of the injection after receiving their vaccinations. Magnetic resonance imaging findings confirmed the formation of an abscess. No other causes of abscess formation were observed. Both patients required surgical intervention and were treated with a course of antibiotics. To our knowledge, this is the first case report to suggest routine vaccinations as a potential cause of abscess formation in premature infants.

## 1. Introduction

The impact of vaccinations has been immeasurable. Since the beginning of the vaccination era, vaccines have protected children from numerous debilitating diseases [1]. Small pox has been eradicated, poliomyelitis is on the verge of extinction, and measles mortality has decreased by 74% [2, 3].

Nowadays a series of routine vaccinations, defined as vaccines that are recommended by the Center for Disease Control and the Advisory Committee on Immunization Practices for all people in the United States, are given at 2 months of age. Under the current immunization guidelines, infants are given their first set of multiple vaccinations at 2 months of age where they are scheduled to receive vaccinations for hepatitis B,* Rotavirus*, diphtheria, tetanus, pertussis,* Haemophilus influenzae* type B, pneumococcus, and inactivated poliovirus [4]. While the benefits of routine infant vaccinations in premature infants have been demonstrated [5–7], there is relatively little data on the dosing of these vaccines in premature infants [8]. This study describes 2 premature infants who develop intramuscular abscesses after receiving their first routine infant vaccination.

## 2. Case Reports

*Patient 1*. A premature twin boy was born at 25.6 weeks who had been receiving inpatient treatment at our institution's Neonatal Intensive Care Unit (NICU) since birth for respiratory distress syndrome. At 2 months of age postpartum, the patient was given his first dose of the routine infant vaccinations, a 0.5 mL dose of Pediarix (DTaP/HepB/Ipv) on 7/10/2015. The following day, the patient developed pain in his right leg that appeared to worsen with extension.

Physical examination revealed a body weight of 2.012 kg (birth weight = 0.921 kg), mild effusion of the right knee, and resistance to passive flexion and extension of the right knee.

Vital signs remained stable and afebrile. No central venous lines were noted on exam.

**Figure 1:**
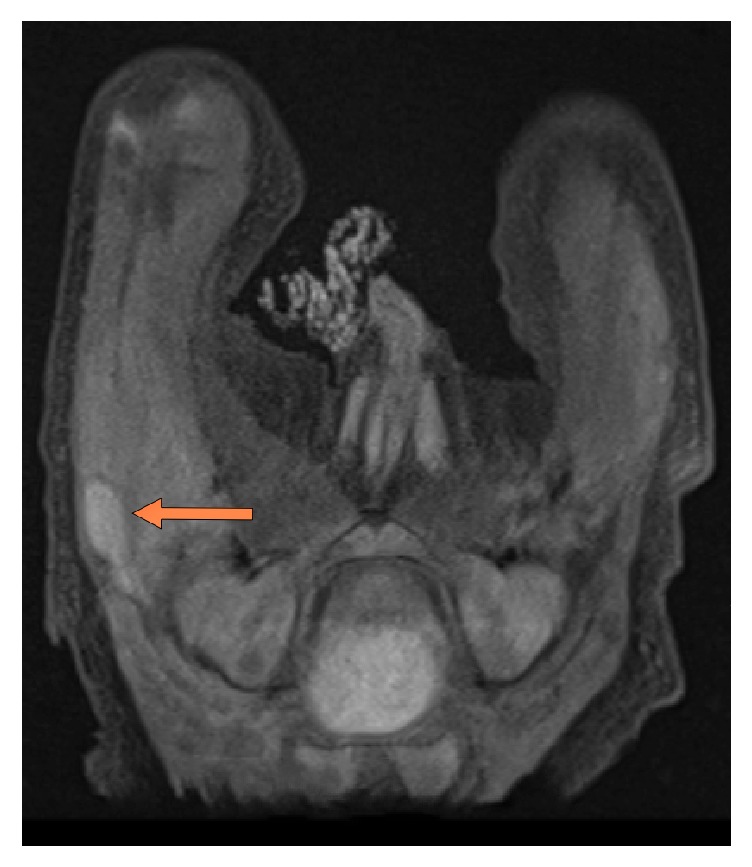
MRI image axial cut demonstrating deep subcutaneous fluid collection with peripheral rim enhancement (arrow) concerning for soft tissue abscess.

Laboratory data showed a white blood count of 18000 cells/mL with 48% neutrophils, hemoglobin of 13.8 g/dL, C-reactive protein of 224 mg/L, and erythrocyte sedimentation rate 47. Ultrasound of the right thigh revealed a 1 × 2 cm collection in the subcutaneous tissues of the right superolateral thigh. MRI of the right femur confirmed a deep subcutaneous fluid collection with peripheral rim enhancement concerning for soft tissue abscess (Figure 1).

Blood cultures were drawn and the patient was started on broad spectrum antibiotics (Vancomycin 20 mg/kg q8h IV, Cefotaxime 50 mg/kg q8h IV). On the fourth day, blood cultures revealed* Staphylococcus aureus* with sensitivity to Oxacillin and Rifampin. As a result, the patient's antibiotic regimen was switched accordingly (Oxacillin 100 mg/kg/day q8h IV, Rifampin 5 mg/kg q12h IV).

Orthopaedic surgery was consulted and the patient was taken to the operating room for incision and drainage of the right thigh abscess. The patient was induced with general anesthesia. An incision was made over the area of the abscess revealing purulent fluid. The abscess was allowed to drain fully and cultures of the fluid were sent off. A temporary Penrose drain was placed and the wound was closed.

Wound cultures from the surgery confirmed* Staphylococcus aureus* with sensitivity to Oxacillin and Rifampin. The patient completed a 28-day course of antibiotics and his lab values returned to normal limits.

*Patient 2*. A premature twin boy was born at 25.4 weeks who had been receiving inpatient treatment at our institution's NICU since birth for respiratory distress syndrome and metabolic acidosis. At 2 months of age postpartum, the patient was given his first dose of the routine infant vaccinations, a 0.5 mL dose of Pediarix (DTaP/HepB/Ipv) on 5/25/2015. One and a half weeks later, the patient was noted to have decreased movement in his right leg.

Physical examination revealed a body weight of 2.047 kg (birth weight = 0.859 kg). His right leg showed decreased spontaneous movement and was held in flexion at rest. The right leg could be passively straightened; however it returned to flexion upon release. Vital signs were significant for a fever of 39.7°C (103.4°F). Central venous line located in the left upper extremity with no signs of erythema or infection.

**Figure 2:**
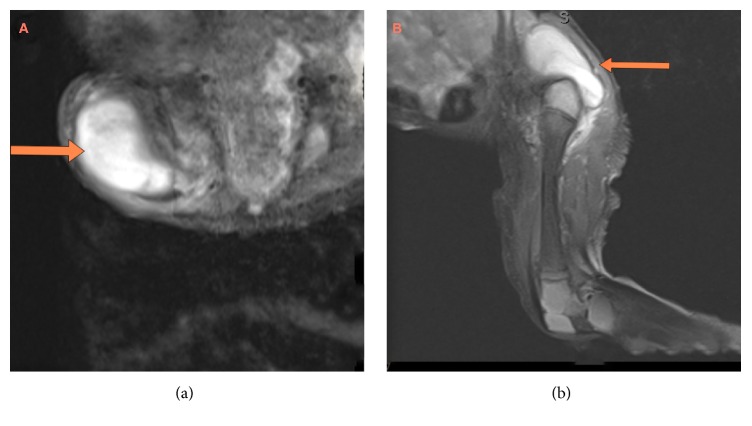
(a) MRI image axial cut demonstrating large subcutaneous fluid collection (arrow) in the thigh and (b) MRI image sagittal cut of the same patient's right hip showing same fluid collection with corresponding joint effusion (arrow) concerning for septic arthritis.

Laboratory data showed a white blood count of 22100 cells/mL with 47% neutrophils, hemoglobin of 13.5 g/dL, and C-reactive protein of 145 mg/L. Ultrasound of the right thigh revealed a subcutaneous fluid collection concerning for an abscess. MRI confirmed the subcutaneous fluid collection in the thigh, along with right hip joint effusion concerning for septic arthritis (Figure 2).

Orthopaedic surgery was consulted and the patient was immediately taken to the operating room for incision and drainage of the abscess along with a joint capsule washout for potential septic joint. The patient was induced with general anesthesia. The soft tissue abscess was aspirated revealing purulent fluid. Approximately 10 mL of viscous pus was removed and sent for cultures. Given the high likelihood of septic joint, a small incision was made in the right anterior crease. The underlying planes were carefully dissected until the cartilaginous femoral head was identified and confirmed with fluoroscopy. The capsule was opened revealing serous fluid that was then sent for culture. The capsule was washed out with sterile solution and closed with a Penrose drain in place.

The patient was initially started on IV Vancomycin and IV Cefotaxime and was eventually switched to IV Vancomycin and IV Cefepime due to concern of hospital acquired infection. Wound cultures grew out methicillin sensitive* Staphylococcus aureus*. As a result, the Vancomycin and Cefepime was discontinued and the patient completed a 28-day course of Oxacillin. The remainder of the course was uncomplicated and the patient's lab values returned to normal limits.

## 3. Discussion

This report describes 2 premature infants who developed an abscess after receiving their first infant vaccination in the form of an intramuscular injection; to our knowledge, these are the first such cases reported in premature infants. The diagnosis of an abscess was confirmed by clinical, radiologic, and laboratory findings.

The rate of complications associated with intramuscular injections has been reported in the past to range from 0.4% to 19.3% [9–11]. When they do occur, they are typically caused by the trauma associated with the injection itself or the irritating properties of the drug [9–11]. Examples of these include leakage or seeping of injected solution from the injection site, bleeding, inadvertent injection of intramuscular medication into arteries or veins, nerve injury, persistent pain, abscess formation, necrosis of the surrounding tissue, scar formation, muscular fibrosis with contracture of joints, and very rare development of malignancy at the injection site [9–11].

When past cases of abscess formation after intramuscular injections have been documented, they have been classified into two groups, sterile abscesses and infectious abscesses [5, 8]. Sterile abscesses are thought to be caused by a foreign body type reaction to an adjuvant or excipient agent used within the vaccines [12, 13] while infectious abscesses are thought to be caused by inoculation of bacteria at the site of injection [9, 12, 14]. Compared to other intramuscular injections, vaccine intramuscular injections can be predisposed to forming a collection due to their adjuvant agents.

In both our cases however, the abscess was confirmed to be of infectious etiology and most likely caused by the inoculation of bacteria during the administration of their DTaP/HepB/Ipv vaccination. Both patients were born prematurely and grew out methicillin sensitive* Staphylococcus aureus* suggesting a nosocomial infection. Both patients required their abscesses to be drained surgically exposing them to the risks associated with surgery.

The question to be considered is if premature infants are at a greater risk of developing abscesses after an intramuscular injection. While the pharmacokinetics of drugs has been extensively tested in adults and children, there is very little data on the pharmacokinetics of drugs within infant populations [8]. Most of the drugs given to infants are currently being dosed based on information extrapolated from adults or older children [15]. Since the neonatal period is a time of rapid physiological change, it is difficult to predict the way a drug is absorbed and distributed within a neonate [8, 16]. However, several studies in the past decade have confirmed previous findings of acceptable safety, immunogenicity, and efficacy of routine infant vaccinations in preterm infants given at a chronological age of 2 months [17].

For our patients, we speculate that the differences in the length of stay of premature neonates ultimately put them at a greater risk for developing an abscess after receiving an intramuscular injection. Compared to full term neonates, premature neonates have longer hospital stays. In a recent study, full term infants were found to have a median length of stay of 1 day from birth to discharge while the median length of stay for late preterm infants (34–36 weeks of gestation) was 2 days [18]. While data is still limited on the average length of stay for severely preterm infants, preterm infants requiring NICU level care generally have longer length of stays compared to other infants.

For the cases presented in this article, their length of stay exceeded 2 months requiring them to receive their 2-month routine vaccinations while hospitalized. We hypothesize that the prolonged length of stay exposed the infants to health care associated pathogens increasing their risk of developing an intramuscular abscess after receiving their vaccination. This hypothesis is further supported by the final wound cultures, which grew out identical methicillin sensitive* Staphylococcus aureus*. When compared to a full term neonate receiving their 2-month routine vaccination in an ambulatory setting, one would expect any infectious complications to be associated with a more ubiquitous group of pathogens.

Routine vaccinations have been extensively studied in premature infants and found to be both safe and effective in generating an immune response [5–7]. However in both cases presented in this article, a premature infant with a prolonged hospital length of stay developed a severe infection following the routine immunization schedule. Therefore, one possible solution would be to perform a root cause analysis to determine the cause of these infections. While any patient with a prolonged length of stay will be exposed to health care associated pathogens, it is important to minimize the possibility of this negative outcome.

## 4. Conclusion

We have described 2 low weight premature infants who developed a pyogenic abscess after receiving their routine infant vaccination. To our knowledge, this is the first report of vaccine associated abscess formation in premature infants, suggesting that vaccination of premature infants with prolonged length of hospital stay could lead to the formation of intramuscular abscesses.
